# Nursing Performance Using Clinical Prediction Rules for Acute Respiratory Infection Management: A Case-Based Simulation

**DOI:** 10.1055/a-2700-7036

**Published:** 2025-10-17

**Authors:** Victoria L. Tiase, Patrice Hicks, Haddy Bah, Ainsley Snow, Devin M. Mann, David A. Feldstein, Wendy Halm, Paul D. Smith, Rachel Hess

**Affiliations:** 1 Department of Biomedical Informatics, School of Medicine, University of Utah, Salt Lake City, Utah, United States; 2 Department of Ophthalmology and Visual Sciences, University of Michigan, Ann Arbor, Michigan, United States; 3 Department of Population Health Sciences, School of Medicine, University of Utah, Salt Lake City, Utah, United States; 4 Department of Population Health and MCIT Department of Health Informatics, NYU Grossman School of Medicine, New York, New York, United States; 5 Department of Medicine, University of Wisconsin School of Medicine and Public Health, Madison, Wisconsin, United States; 6 University of Wisconsin-Madison School of Nursing, Madison, Wisconsin, United States; 7 Department of Family Medicine and Community Health, University of Wisconsin School of Medicine and Public Health, Madison, Wisconsin, United States

**Keywords:** antimicrobial stewardship, clinical decision support, clinical practice guideline, clinical informatics, nurse

## Abstract

**Background:**

Overuse and misuse of antibiotics is an urgent health care problem and one of the key factors in antibiotic resistance. Validated clinical prediction rules have shown effectiveness in guiding providers to an appropriate diagnosis and identifying when antibiotics are the recommended choice for treatment.

**Objectives:**

We aimed to study the relative ability of registered nurses using clinical prediction rules to guide the management of acute respiratory infections in a simulated environment compared with practicing primary care physicians.

**Methods:**

We evaluated a case-based simulation of the diagnosis and treatment for acute respiratory infections using clinical prediction rules. As a secondary outcome, we examined nursing self-efficacy by administering a survey before and after case evaluations. Participants included 40 registered nurses from three academic medical centers and five primary care physicians as comparators. Participants evaluated six simulated case studies, three for patients presenting with cough symptoms, and three for sore throat.

**Results:**

Compared with physicians, nurses determined risk and treatment for simulated sore throat cases using clinical prediction rules with 100% accuracy in low-risk sore throat cases versus 80% for physicians. We found great variability in the accuracy of the risk level and appropriate treatment for cough cases. Nurses reported slight increases in self-efficacy from baseline to postcase evaluation suggesting further information is needed to understand correlation.

**Conclusion:**

Clinical prediction rules used by nurses in sore throat management workflows can guide accurate diagnosis and treatment in simulated cases, while cough management requires further exploration. Our results support the future implementation of automated prediction rules in a clinical decision support tool and a thorough examination of their effect on clinical practice and patient outcomes.

## Background and Significance

It is estimated 30 to 50% of antibiotic prescribing occurring outside of hospitals in the United States is unnecessary. 1 2 While antibiotics used for treatment of bacterial infections has increased life expectancy across the world, 3 4 antibiotic overuse can increase the risk of antibiotic resistance. 5 6 More than 2.8 million antibiotic-resistant infections occur in the United States each year, and more than 35,000 people die as a result. 7

Health care providers inappropriately prescribe antibiotics for many reasons, including misdiagnosis of an infection as bacterial or satisfying a patient's desire for antibiotics as treatment. 8 Clinical prediction rules (CPRs) are evidence-based tools used at the point of care that can help health care providers with evidence-based clinical decisions such as determining when antibiotics are appropriate for treatment or when further testing for an infection is unnecessary. 9 10 CPRs are developed using systematic reviews of the state of the science and their creation may be funded by agencies such as the Agency for Healthcare Research and Quality to prevent harm. 11

When integrated into electronic health records (EHRs), CPRs use data from a patient's past medical history, physical examination, current symptoms, and laboratory results to determine the individual patient's risk of having a disease. CPRs also take the form of clinical decision support rules that prompt providers to review guidance within their workflows. Although well-validated, CPRs remain underused, suggesting issues with adoption, usability, or poor integration into clinical workflows. 12

Previous research has shown that physicians do not widely adopt CPRs for patients with acute respiratory infections (ARIs), specifically those presenting with a sore throat and cough. 13 14 15 One alternative to targeting CPRs to physicians is to target nurses. Nurses possess a combination of clinical expertise, communication skills, patient-centered care, and a commitment to evidence-based practice, making them well-suited for using CPRs. 16 They play a vital role in facilitating the application of CPRs in clinical settings, contributing to more informed and patient-centered decision-making. 17 For example, if a nurse sees a patient with a chief complaint of sore throat presenting without a fever and mild symptoms, it is within the nurse's scope of practice to provide and advise on supportive treatment (rest, hydration, and over-the-counter remedies) depending on the state and facility protocols. Supporting nurses to use CPRs in the evaluation of patients with sore throat and cough symptoms may overcome barriers to physician-focused interventions and potentially lead to a reduction in inappropriate antibiotic prescribing for ARIs.

Nurses have effectively used care protocols to improve ambulatory care across a number of chronic diseases, including diabetes, hypertension, anticoagulation (e.g., coumadin clinic), and congestive heart failure. 18 Randomized controlled trials comparing nurses to physicians for treatment of acute minor illness found nurses took slightly more time with patients but had equivalent symptom resolution, with small but significant increases in patient satisfaction measures. 19 20 Additionally, the largest study examining nursing treatment for acute minor illnesses reported a positive outcome for patient experience. 21 In Spain, nurses in primary care clinics were trained to use algorithms for 16 acute minor illnesses and were able to resolve 63% of the 1.2 million visits without consulting a physician. 21 Previous research supports our hypothesis that efforts to empower nurses to deliver high-quality, supported care to patients with both sore throat and cough symptoms may not only influence prescribing, but also affect overall patient care, patient satisfaction, and nurse satisfaction. 22

## Objective

The purpose of this study was to determine whether nurses could identify appropriate treatment options for simulated patients with cough and sore throat symptoms using historically physician-oriented CPRs to guide treatment. Specifically, we wanted to determine the relative ability of nurses to appropriately use CPRs to guide management of ARIs in a simulated environment as preparation for a large-scale clinical trial. Using a validated, nursing-specific self-efficacy scale, we also sought to measure the levels of self-efficacy, or belief in one's own capabilities to successfully perform tasks, for the nurses using CPRs. 23 Our main goal was to test key assumptions in the deployment of nurse-facing CPRs in support of a nurse-driven electronic health record (EHR), ARI management workflow designed to reduce inappropriate antibiotic prescribing.

## Methods

### Participants and Recruitment

**Fig. 1:**
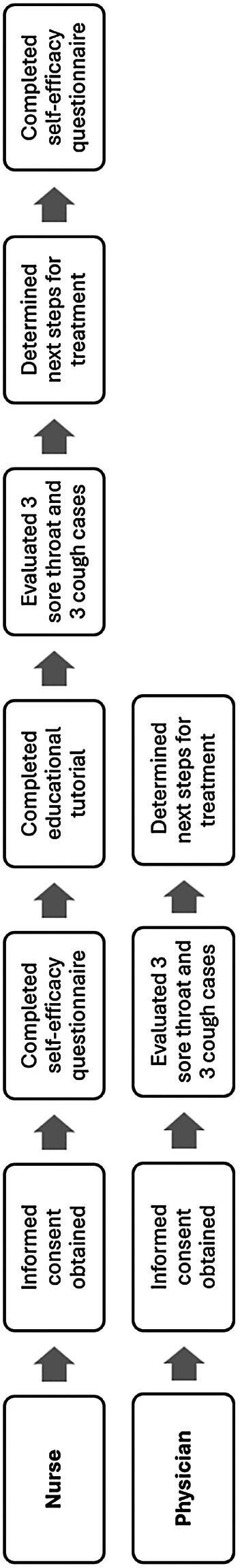
Nurses and physicians study processes.

We recruited registered nurses over the age of 18 from NYU Langone, University of Wisconsin, and University of Utah academic medical centers currently working in a clinical setting to participate in a case-based simulation. Nurse practitioners and other APRNs were excluded from participating in the study. Using snowball sampling, the study staff approached nurses during staff meetings at their corresponding clinics' settings. In addition, potential participants were contacted via email to see if they were eligible and interested. Flyers were posted on the nursing units of the respective institutions. If a participant was deemed eligible, they were sent an email containing a link to a Research Electronic Data Capture (REDCap) enrollment form. 24 25 Given the number of cough and sore throat cases per month, we calculated a sample size of 40 nurses was needed for recruitment. We also recruited practicing primary care, internal medicine physicians with experience in treating ARIs drawn from the leadership team at the study sites to serve as a comparison group. Nurses were compensated with a $25.00 gift card for participating in the research study, and we did not obtain approval to compensate physicians. All study procedures were reviewed and approved by the University of Utah Institutional Review Board, and all participants were appropriately consented to participate ( Fig. 1 ).

### Study Procedures

After the nurses entered their unique code to participate in the study, we collected demographic information, including their age, sex, highest degree, and number of years of clinical experience. Participants were also asked to complete a short self-efficacy questionnaire, which served as a baseline. We used a modified version of the Self-Efficacy in Clinical Performance instrument developed and validated for nursing students, which resulted in 12 questions ( Supplementary Appendix 1 ). 26 Questions were scored using a scale of zero (cannot do at all) to 100 (highly certain I can do). After completing the simulated cases, nurses were asked to complete the questionnaire a second time, separately for cough and sore throat cases. This study leveraged the ARI CPRs used in prior studies and considered appropriate for application in clinical practice, given the high degree of confidence in their accuracy. 27 28 29 In addition, the case development and scoring rubric were refined by three physicians with expertise in antibiotic stewardship. Each of these CPRs provides a level of risk of disease (low, medium, and high) aligning with guideline-based clinical recommendations. 28 As part of the sore throat CPR used to predict risk of strep throat: (1) scores of zero to one represent low risk with no antibiotic prescribing or further testing recommended; (2) scores of two to three represent moderate risk with suggested rapid strep testing; and (3) scores of four or five represent high risk with antibiotic treatment recommended after a rapid strep testing confirmation. 30 31 The cough CPR used to predict risk of pneumonia consisted of: (1) scores of zero to one as low risk requiring no antibiotic prescribing or further testing; (2) scores of two to three are considered medium risk with the recommendation of chest X-ray (CXR) and antibiotics if the CXR is abnormal; and (3) scores of four to five represent high risk with a recommendation of antibiotics after a confirmed abnormal CXR ( Table 1 ). 28 32 33

**Table 1:** Clinical prediction rules for cough and sore throat

Case type	Questions	Scoring rubric
Sore throat	Fever by history	Yes = 1, No = 0
	Absence of cough	Yes = 0, No = 1
	Tender anterior cervical adenopathy	Yes = 1, No = 0
	Tonsillar excaudate	Yes = 1, No = 0
Cough	Heart rate > 100 beats per minute	Yes = 1, No = 0
	Temperature > 37.8°C or 100°F	Yes = 1, No = 0
	History of asthma	Yes = 0, No = 1
	Presence of rales	Yes = 1, No = 0
	Decreased breath sounds	Yes = 1, No = 0

Nurse participants completed an educational tutorial to review the assessment and treatment for patients with pneumonia and strep throat prior to conducting the simulation. Physicians were excluded from the educational tutorial portion of the study since they received extensive training on clinical practice guidelines as part of their medical education and regular use in practice. The educational tutorial was created by a clinical nursing instructor and included information about strep throat and pneumonia through digital learning aids. These learning aids included an audio recording of breath sounds as well as photographs of the throat depicting indicators for the presence and absence of both pneumonia and strep throat. The nurses were asked to answer questions pertaining to the material reviewed during the tutorial. We required the nurses to answer all questions correctly before proceeding to the next step. If a question was answered incorrectly, the participant was able to review the correct answer with rationale. The educational tutorial took approximately 20 to 25 minutes to complete.

Both nurse and physician participants were asked to diagnose a total of six simulated cases using the CPRs. The cases consisted of three patients with complaints of a cough and three with complaints of a sore throat. Participants were asked to watch a short video corresponding to each of the six cases ( Supplementary Appendices 2 and 3 ). After the video, each participant completed the sore throat and cough CPR questions for the six cases. We presented the participants with their final risk score, and then asked the participants to select the next steps for treatment, which could either be based on their identified risk score or their own clinical judgement. The participants' treatment selections were recorded electronically. Since deception was not a part of the simulation, debriefing sessions were not conducted as a part of this study.

**Table 2:** Participant demographics

		Nurses ( *n* = 40)		Physicians ( *n* = 5)	
Measure	Item	Count	%	Count	%
Sex	Female	38	95.0	2	40
	Male	2	5.0	3	60
Experience	<1 y	3	7.5	0	0
	1–5 y	4	10	1	20
	6–9 y	11	27.5	0	0
	≥10 y	22	55.0	4	80
Education	RN, associate's degree	19	47.5	0	0
	RN, bachelor's degree	20	50.0	0	0
	RN, clinical nurse coordinator	1	2.5	0	0
	Doctor of medicine	0	0	5	100

Abbreviation: RN, registered nurse.

**Table 3:** Risk identification and treatment decision correctness

Case type		Nurses ( *N* = 40)	Physicians ( *N* = 5)
Cough	Risk		
	Low	30 (75.0%)	5 (100%)
	Medium	30 (75.0%)	5 (100%)
	High	37 (92.5%)	5 (100%)
	Treatment		
	Low—no treatment/testing	31 (77.5%)	5 (100%)
	Medium—chest X-ray	30 (75.0%)	5 (100%)
	High—treat with antibiotics	35 (87.5%)	5 (100%)
Sore throat	Risk		
	Low	40 (100%)	4 (80.0%)
	Medium	40 (100%)	2 (40.0%)
	High	39 (97.5%)	5 (100%)
	Treatment		
	Low—no treatment/testing	40 (100%)	4 (80.0%)
	Medium—rapid strep, treat if +	39 (97.5%)	2 (40.0%)
	High—treat with antibiotics	38 (95.0%)	5 (100%)

### Data Analysis

Descriptive statistics were compiled for the participant demographics. Results of the treatment decisions were summarized by type of risk and selected next steps for treatment. Nurse self-efficacy questionnaire responses for pre- and postcase simulations were aggregated by the average response score for each question and contributed to an overall score for pre- and postresponses. Agreement within physicians and nurses and between physicians and nurses was assessed using Fleiss' kappa, a statistic used to determine interrater reliability when there are three or more raters. 34 35 We interpreted the agreement results using the Landis and Koch interpretation guidelines. 36 All analyses were carried out using R statistical software version 4.0.0 (R Foundation for Statistical Computing, Vienna, Austria) using the interrater reliability and agreement pack in R. 37

## Results

Of the 40 nurses who agreed to participate, 95% were female and 55% had ≥10 years of nursing experience. There was only one nurse with the title of clinical nurse coordinator, whereas all other study participants were staff nurses. Of the five physicians who agreed to participate in the study, 40% were female and 80% ≥10 years of experience ( Table 2 ).

### Treatment Decisions

In the evaluation of cough cases, all physicians agreed on all risk levels and the next steps for treatment. For nurses, 75% correctly identified the risk for low- and medium-risk cases and 92.5% correctly identified the high-risk cough cases. For sore throat cases, all the physicians identified high-risk correctly, less than half identified medium-risk (40%), and most identified low-risk (80%). All physicians correctly determined treatment with antibiotics as the next step for high-risk sore throat cases. For the medium-risk sore throat cases, some of the physicians correctly identified the need to conduct a rapid strep screening first and then treat if positive.

Nurses outperformed physicians in the identification of the accurate risk level for sore throat cases. In sore throat cases, nurses identified the correct risk for all the low- and medium-risk cases. Only one nurse incorrectly determined the high-risk sore throat case. All nurses agreed on the low-risk sore throat treatment. One nurse did not select the correct treatment for the medium-risk sore throat case. Two nurses did not select the correct treatment for the high-risk sore throat case by underprescribing—or omitting a medication that is indicated for treatment, without a reason for not prescribing it. Overall, physicians correctly identified all cough cases; however, nurses outperformed the physicians across the sore throat cases ( Table 3 ).

### Agreement

For the responses pertaining to risk and treatment of cough cases, there was almost perfect agreement (K = 1.000 [95% CI: 0.999, 1.000]) among the physicians. For the sore throat cases, there was slight or poor physician agreement for both risk (K = 0.041 [95% CI: −0.410, 0.492]) and treatment (K = 0.041 [95% CI: −0.410, 0.492]), characterized by undertesting in the medium-risk cases and overprescribing in the low-risk cases.

The nurses' responses showed moderate agreement of both risk (K = 0.537 [95% CI: 0.487, 0.587]) and treatment (K = 0.506 [95% CI: 0.456, 0.556]) of cough cases. In the sore throat cases, there was almost perfect agreement across the nurses' responses for both risk (K = 0.975 [95% CI: 0.925, 1.000]) and treatment (K = 0.926 [95% CI: 0.876, 0.976]) of cases. When observing the responses for determination of risk between nurses and physicians, we found there was moderate agreement between the two participant groups for the risk of cough cases (K = 0.512 [95% CI: 0.375, 1.000]). However, in sore throat cases, there was slight agreement between nurses and physicians when determining risk (K = 0.125 [95% CI: 0.000, 0.500]).

We found similar results for the treatment of both cough cases (K = 0.500 [95% CI: 0.375, 1.000]) and sore throat cases (K = 0.188 [95% CI: 0.000, 0.512]; Table 4 ). Within the sore throat cases, there was slight agreement between physicians and nurses and also slight agreement among the nurses on the next steps for treatment. Across the physicians, we found both over- and underprescribing. There was moderate agreement between physicians and nurses within the cough cases.

**Table 4:** Degree of agreement for case risk and treatment

Physician agreement	Risk kappa values	Treatment kappa values
Cough cases	1.000 (95% CI: 0.999, 1.000)	1.000 (95% CI: 0.999, 1.000)
Sore throat cases	0.041 (95% CI: −0.410, 0.492)	0.041 (95% CI: −0.410, 0.492)
Nurse agreement		
Cough cases	0.537 (95% CI: 0.487, 0.587)	0.506 (95% CI: 0.456, 0.556)
Sore throat cases	0.975 (95% CI: 0.925, 1.000)	0.926 (95% CI: 0.876, 0.976)
Nurses physicians agreement		
Cough cases	0.512 (95% CI: 0.375, 1.000)	0.500 (95% CI: 0.375, 1.000)
Sore throat cases	0.125 (95% CI: 0.000, 0.500)	0.188 (95% CI: 0.000, 0.512)

Abbreviation: CI, confidence interval.

### Self-Efficacy Outcomes

For both cough and sore throat cases, the mean scores for self-efficacy of the nurse participants marginally increased from baseline results, collected before training, to the results that were collected after each simulated case evaluation. In cough cases, the self-efficacy mean score increased from 89.47 (standard deviation [SD] = 11.41) pretraining to 90.64 (SD = 10.62) posttraining. In sore throat cases, the pretraining mean score was 89.74 (SD = 12.06) and the posttraining mean score was 93.65 (SD = 08.66).

## Discussion

In this simulation study using CPRs for patients presenting with cough and sore throat, we found nurses were more accurate in determining the diagnosis and treatment of sore throat cases, whereas physicians were more accurate in determining the diagnosis and treatment of cough cases. The difference between cough and sore throat may be due to the additional steps needed in a cough assessment and fewer in determining sore throat risk. Previous research conducted within nurse triage clinical care found nurses can provide both effective and safe treatment to patients with sore throat symptoms, and our study further supports this finding. 18 38

Since some nurses may not be familiar with using CPRs in patient care, nurses may require additional training. We believe this can be accomplished by creating additional simulated patient cases as a means of practice during orientation and ongoing. Continuing education is standard practice in health care professions and simulated patient cases within nursing education have proved to be effective. 39 40 Given that nurses were accurate in both the identification of risk and treatment of the simulated sore throat cases, providing the opportunity to treat sore throat cases using CPRs within the context of a clinical trial may be a worthwhile next step.

Surprisingly, we found poor agreement amongst the physicians in terms of risk and treatment of sore throat cases. This may be because the physicians were not solely influenced by clinical decision rules and continued to rely on clinical experience, rather than using consensus guidelines. In addition, organizational mandates on improved patient satisfaction rates might cause physicians to appease patients expecting a strep test and antibiotics whether or not it's recommended. 41 Previous research has indicated that once physicians establish their antibiotic behavior, it is difficult to change. 32 33

We suspected physicians and nurses using the same guidelines would agree with each other in terms of risk and treatment for cough and sore throat cases. Poor agreement between the sore throat cases among both groups, and only a moderate agreement between the two groups in the cough cases was a surprising result. This finding may suggest differences in clinical judgment, training, or experience. Future work to correlate demographic factors with the individual responses or examine agreement across cohorts with similar training and experience may provide insights.

The self-efficacy responses of the nurses were slightly higher after completing the case simulation in both cough and sore throat cases. By providing nurses with the tools to work at the top of their license, such as the CPRs used in this study, it often leads to increased job satisfaction, which is highly correlated with self-efficacy. 40 Studies have also linked higher self-efficacy to increased motivation and positive attitudes. 42 Additionally, nurses are more engaged and motivated when they can use their skills and knowledge to their fullest extent, which can contribute to reduced burnout and turnover rates. 40 However, we did not explore this correlation fully with this study. As we investigate the use of CPR-adjusted workflows inserted in nursing practice in the future, we plan to gain a greater understanding of nursing beliefs and perspectives on self-efficacy and correlate aspects with retention.

Findings from this study have implications for influencing clinical care decisions and enhancing the understanding of the role nurses may play in the management of antibiotic stewardship. Nursing involvement in CPR use may be helpful in optimizing health care delivery, improving patient outcomes, and addressing the challenges of the health care system. It allows for a more efficient allocation of health care resources, may enhance patient safety and satisfaction, and may promote cost-effective, evidence-based care. By including CPRs as part of EHR decision support tools, we can expand our understanding of decision support needs for nursing practice. Additionally, such tools can be a key component of the evolving health care landscape, particularly as health care systems seek innovative ways to address shortages of health care providers and provide high-quality care to a growing and diverse patient population.

Limitations of this study include: the small sample size, only academic medical center settings, and the experience level of participants. Future research should include a larger sample of nurses and physicians with varied levels of experience. We acknowledge that performance biases may exist, since we did not exclude participants based on experience level. Since we recruited participants from three geographically diverse academic medical center settings who may have benefited from specific forms of training and experiences, it will also be important to replicate this work with participants from other health care settings, such as community settings. Although our initial focus was on nurses and physicians, future research may benefit from the inclusion of other health care team members who are licensed to diagnose and treat sore throat and cough cases, such as physician assistants and advanced practice nurses. Exploring an interdisciplinary approach may increase team-based CPR use and uncover unrealized benefits. In addition, we did not assess the clinical reasoning used to determine risk and treatment. Future research may also benefit from the inclusion of the qualitative method during case-based evaluations, so individually, tailored training approaches may be implemented.

## Conclusion

Nurses more accurately determined risk and treatment for simulated sore throat cases using CPRs compared with physicians. Our findings could be used to inform future research in automating CPRs within EHRs, the use of CPRs by other health care professionals, and a thorough understanding of nurse perceptions and beliefs while using decision support tools in practice. Equipping and training nurses to use CPRs for the diagnosis and treatment of patients with cough and sore throat symptoms has the potential to decrease antibiotic prescribing and influence antibiotic stewardship. In creating efficiencies for primary care, we may, in turn, increase access to care for patients in general and specifically for underserved populations.

## Clinical Relevance Statement

Overall, this study underscores the potential benefits of using CPRs in nursing practice to improve patient outcomes, enhance health care delivery, and support antibiotic stewardship efforts. By accurately diagnosing and treating sore throat cases, nurses can help reduce unnecessary antibiotic prescriptions, which is crucial in combating antibiotic resistance. Moreover, implementing CPRs in clinical workflows can create efficiencies in primary care, potentially increasing access to care for patients. The findings support the future integration of automated CPRs and EHRs in a clinical trial, which may lead to more consistent and evidence-based clinical decision-making.

## Multiple-Choice Questions

1. Which of the following statements is true regarding the use of clinical prediction rules (CPRs) by nurses and physicians in the study?
  - Nurses were more accurate in diagnosing and treating cough cases compared with physicians.
  - Physicians showed higher self-efficacy after using CPRs in simulations.
  - Nurses were more accurate in diagnosing and treating sore throat cases compared with physicians.
  - There was no difference in the accuracy of diagnosing and treating sore throat cases between nurses and physicians.
  **Correct Answer** : The correct answer is option c. As per Table 3 , nurses were more accurate in diagnosing and treating. For diagnosing, low and medium risk was identified by 100% of the nurses, whereas physicians were only 80 and 40%, respectively. In treating low and medium risk, nurses identified the correct treatment 100 and 97.5% of the time compared with 80 and 40% for physicians.
2. What potential benefit of using CPRs in nursing practice was highlighted in the study?
  - Increased antibiotic prescribing.
  - Decreased job satisfaction among nurses.
  - Decreased access to care for underserved populations.
  - Reduced burnout and increased job satisfaction among nurses.
  **Correct Answer** : The correct answer is option d. Nurses reported minimal increased self-efficacy after using CPRs in simulations. Higher self-efficacy is linked to greater job satisfaction and reduced burnout, which can improve overall nurse retention and performance.
3. What is one of the implications of the study for future research and clinical practice?
  - Discontinuing the use of CPRs in clinical decision support tools.
  - Automating CPRs within electronic health records.
  - Reducing the role of nurses in primary care.
  - Increasing reliance on clinical experience over evidence-based guidelines.
  **Correct Answer** : The correct answer is option b. The findings support the future integration of automated CPRs within electronic health records and their use by nurses in clinical decision support workflows. This can lead to more consistent and evidence-based clinical decision-making.
