# Supplementary material for: Nursing Performance Using Clinical Prediction Rules for Acute Respiratory Infection Management: A Case-Based Simulation
Source: Appl Clin Inform. 2025 Oct 17;16(5):1359–67. doi: 10.1055/a-2700-7036 (PMC12534126; doi:10.1055/a-2700-7036)
Supplement: Supplementary file 1 — Supplementary Material [file 10-1055-a-2700-7036_27253407.pdf]

Clinical Prediction Rules ARI

Supplementary Appendix 1 Self-efficacy questions

Question type	Question
Cough	Collect significant data by performing physical assessment.
	Collect relevant data by taking a patient's history.
	Collect data within the time available.
	Collect objective data related to patient health condition.
	Collect subjective data related to patient health condition.
	See relationships among pieces of data that were collected from a variety of sources.
	Document collected data based on patient health condition.
	Analyze collected data based on patient health condition.
	Identify patient's strengths in the care process.
	Identify patient's concerns in the care process.
	Set priorities for patient's problems based on patient health condition.
	I am confident in my ability to evaluate and treat a patient with a cough.
Sore throat	Collect significant data by performing physical assessment.
	Collect relevant data by taking a patient's history.
	Collect data within the time available.
	Collect objective data related to patient health condition.
	Collect subjective data related to patient health condition.
	See relationships among pieces of data that were collected from a variety of sources.
	Document collected data based on patient health condition.
	Analyze collected data based on patient health condition.
	Identify patient's strengths in the care process.
	Identify patient's concerns in the care process.
	Set priorities for patient's problems based on patient health condition.
	I am confident in my ability to evaluate and treat a patient with a sore throat.

Supplementary Appendix 2 Simulated cases

Case type	Patient description
Low risk sore throat	• 40-year-old<br>• No past medical history<br>• No medications/allergies<br>• Vitals include a temperature of 99.0 degrees Fahrenheit, blood pressure of 125/85 and a heart rate of 80<br>• Visiting for an acute visit for a sore throat
Medium risk sore throat	• 40-year-old No past medical history<br>• No medications/allergies<br>• Vitals include a temperature of 101.0 degrees Fahrenheit, blood pressure of 125/85 and a heart rate of 80<br>• Visiting for an acute visit for a sore throat
High risk sore throat	• 40-year-old<br>• She has no past medical history<br>• She takes no medications and has no allergies<br>• Vitals include a temperature of 101.0 degrees Fahrenheit, blood pressure of 120/80 and a heart rate of 90

Applied Clinical Informatics | DOI 10.1055/a-2700-7036 | © The Author(s).

Clinical Prediction Rules ARI

	• Visiting for an acute visit for a sore throat
Low risk cough case	• 40-year-old<br>• No past medical history<br>• No medications/allergies<br>• Vitals include a temperature of 99.5.0 degrees Fahrenheit, blood pressure of 125/85 and a heart rate of 80<br>• Pulse O2 sat: 99%<br>• Visiting for an acute visit for a cough
Medium risk cough case	• 40-year-old<br>• Patient has Hypertension<br>• Patient is taking amlodipine 10mg, No allergies<br>• Vitals include a temperature of 101.2 degrees Fahrenheit, blood pressure of 120/85 and a heart rate of 92<br>• Pulse O2 sat: 96%<br>• Visiting for an acute visit for Hypertension
High risk cough case	• 40-year-old<br>• She has no past medical history<br>• She takes no medications and has no allergies<br>• Vitals include a temperature of 101.4 degrees Fahrenheit, blood pressure of 120/85 and a heart rate of 105<br>• Pulse O2 sat: 96%<br>• Visiting for an acute visit for a cough

Supplementary Appendix 3 Examination findings

Type of case	Exam findings
Low risk sore throat	• Vitals per chart<br>• HEENT: Erythematous oropharynx with evidence of postnasal drip; No tonsillar exudate; Non-tender. Anterior cervical lymphadenopathy; No other lymphadenopathy;<br>• Ears with normal canals and tympanic membranes clear with no bulging.<br>• Lungs: Clear to auscultation<br>• Skin: No rashes
Medium risk sore throat	• Vitals per chart<br>• HEENT: Erythematous oropharynx with no tonsillar exudate; Very tender anterior cervical lymph nodes with no fluctuance. Minimal non-tender posterior lymphadenopathy with no other lymphadenopathy.<br>• Ears with normal canals and tympanic membranes clear with no bulging.<br>• Lungs: Clear to auscultation<br>• Skin: no rashes
	• Vitals per chart

Applied Clinical Informatics | DOI 10.1055/a-2700-7036 | © The Author(s).

Clinical Prediction Rules ARI

High risk sore throat	• HEENT: Erythematous oropharynx with some tonsillar exudate; Very tender anterior cervical lymph nodes with no fluctuance. Mildly tender posterior lymphadenopathy with no other lymphadenopathy.<br>• Ears with normal canals and tympanic membranes clear with no bulging.<br>• Lungs: Clear to auscultation<br>• Skin: no rashes
Low risk cough cases	• Vitals per chart –T: 99.5 HR: 80 Pulse O2 Sat: 99%<br>• HEENT: Erythematous oropharynx without exudate; Diffuse, nontender anterior and posterior cervical lymphadenopathy. No other lymphadenopathy.<br>• Ears with normal canals and tympanic membranes clear with no bulging.<br>• CVS: RRR. Strong pulses<br>• Lungs: Clear to auscultation<br>• Skin: no rashes
Medium risk cough case	• Vitals per chart T:101.2, HR: 92, Pulse O2 Sat: 96%<br>• HEENT: Erythematous oropharynx with some evidence of postnasal drip; No tonsillar exudate; Non-tender anterior cervical lymphadenopathy; No other lymphadenopathy;<br>• Ears with normal canals and tympanic membranes clear with no bulging.<br>• CVS: Tachycardia regular. Strong pulses<br>• Lungs: Some crackles at the L lower lung field. No egophony, no dullness to percussion<br>• Skin: Warm, No rashes
High risk cough case	• Vitals per chart T:101.4, HR: 105, Pulse O2 Sat: 96%<br>• HEENT: Oropharynx a little erythematous without exudate; Diffuse nontender anterior and posterior cervical lymphadenopathy. No other lymphadenopathy.<br>• Ears with normal canals and tympanic membranes clear with no bulging.<br>• CVS: Tachycardia regular. Strong pulses<br>• Lungs: Crackles L lower lung posteriorly. Egophony L lower lung. No dullness to percussion.<br>• Skin: Warm. No rashes

Applied Clinical Informatics | DOI 10.1055/a-2700-7036 | © The Author(s).
